# Intensity of Energy Drink Use Plus Alcohol Predict Risky Health Behaviours among University Students in the Caribbean

**DOI:** 10.3390/ijerph15112429

**Published:** 2018-11-01

**Authors:** Sandra D. Reid, Jannel Gentius

**Affiliations:** 1 Psychiatry Unit, Department of Clinical Medical Sciences, The University of the West Indies, St. Augustine, Trinidad and Tobago; 2 Department of Behavioural Sciences, The University of the West Indies, St. Augustine, Trinidad and Tobago; jannelphilip@yahoo.com

**Keywords:** energy drinks, alcohol, AmEDs, risky behaviours, health behaviours, Trinidad and Tobago, Caribbean

## Abstract

The relationship between energy drink (ED) use and risky behaviours has not been explored in the Caribbean, where youth risky behaviours are prevalent. This paper examines the relationship between ED use and risky behaviours and the moderating effect of gender among 1994 undergraduate students in Trinidad and Tobago. Analyses examined the association between ED use and risky behaviours, and the significant predictors of risky behaviours. Analysis of variance determined whether there were differences in risky behaviours between those who used only alcohol or EDs, both alcohol and EDs, alcohol mixed with EDs and neither alcohol nor EDs; and the difference between those with high and low intensity of ED use. In both males and females current use of energy drinks was positively associated with harmful substance use, risky sexual behaviours, and risky non-sexual behaviours, more strongly for males. The strongest predictor of risky behaviours was gender. Males consuming both alcohol and EDs, independently or mixed, were most likely to have risky behaviours. Consistent with previous reports, harmful alcohol use and other risk taking health behaviours appear to be predicted by a combination of high intensity use of EDs and alcohol. In countries like Trinidad and Tobago where violence, alcohol use disorders, STI/HIV infection and teenaged pregnancy are prevalent, the assessment of consumption of EDs and alcohol may be an important potential predictor of health compromising behaviours.

## 1. Introduction

The use of energy drinks is prevalent among college students and young adults in many countries [1,2,3,4,5,6,7]. With their ongoing popularity and minimal regulatory policies, there continues to be concern over the potentially harmful impact of excessive caffeine intake on general health when these drinks are consumed [8,9,10,11]. Researchers have however placed most attention on the use of energy drinks in combination with alcohol, a practice considered dangerous by some [12,13]. The danger is perceived as a direct result of the interacting pharmacologic effects of the drugs [14,15,16,17,18,19]. Specifically, it has been reported that the stimulant effect of caffeine in energy drinks allows individuals to consume dangerous quantities of alcohol while still being alert and awake [14,15,16]. Users of the combination appear to develop tolerance to the internal cues of intoxication resulting in a false sense of security [17]. The alcohol and energy drink combination thus impairs accurate judgment of the level of intoxication, leading to “wide-awake drunkenness” but does not decrease alcohol-related impairment [12,18,19]. This results in more alcohol-related physical consequences, including increased risk of drunk driving, alcohol-related injury, alcohol poisoning, dehydration and death [12,15,17,20]. Students who consumed pre-mixed alcohol and energy drink combinations (AmEDs) had twice the odds of experiencing one or more negative consequences from AmED use while driving [16,20] and are reported as having greater risk of alcohol related violence, injury, and sexual assault [12,15,21].

There have also been reports that consumers of AmEDs engage in more binge drinking [21], drink faster that those who drink alcohol alone [22], and have greater desire to drink [23,24,25], with an increased risk of developing an alcohol use problem [26]. Even in a community sample, users of AmEDs were more likely to binge drink and engage in hazardous drinking than non-users [27].

Alcohol related harm is not the only area of risky behaviours that has been explored. Snipes and Benotsch [28] found that AmED users were significantly more likely to report marijuana, cocaine and ecstasy use, illicit prescription drug use, and to engage in high risk sexual behaviours. The debate continues as to whether the risky behaviours associated with AmEDs are due to the alcohol, rather than the mix, or to underlying personality traits [29,30,31].

While a lot of attention has been focused on AmEDs and comparing alcohol related risk between those who mix alcohol and EDs and those who use alcohol alone [22,32], fewer researchers have looked at the relationship between pure ED use and risky behaviours. Yet there is evidence to suggest that the use of EDs without alcohol is also associated with several of the same behaviours of public health concern. In fact, Peacock et al. [31] found lower rates of self-reported risk taking among persons consuming AmEDs compared to those using energy drinks alone. Energy drink use has been consistently linked to heavy episodic drinking and subsequent serious alcohol-related consequences such as sexual assault, driving under the influence and increased risk of alcohol dependence [12,33,34,35,36]. When compared to energy drink non-users, those who drank energy drinks were also found to have greater involvement in other drug use with higher levels of sensation seeking and risk taking [4,6,32,33,34,35]. 

Both Miller [33] and Kaminer [35] reported a positive association between risk taking behaviours such as tobacco, marijuana and illicit drug use, sexual risk taking, fighting and seatbelt omission and the frequency of energy drink consumption, while Woolsey et al. [37] and Miller [38] further identified energy drink use as a predictor of risk taking behaviour and identity. This supports the review of Verster et al. [30] who suggested that a personality with higher levels of risk taking behaviour may be the reason for increased alcohol and drug use among users of AmEDs.

Studies on energy drink use among tertiary level students have consistently shown that ED use is more common and occurs to a greater extent among males than females [4,6,7,34,38], with few exceptions [1]. However, studies have generally overlooked whether the relationship between energy drink use and risky behaviours varies by gender. Mostly the report is again that males who drink energy drinks are more likely to have associated risky behaviours [7,33,38]. Very few researchers have specifically examined the degree to which gender mediates the relationship between energy drink use and its behavioural outcomes. In 2008 when looking at the relationship between energy drink use and problem behaviour outcomes, Miller examined the moderating effect of race, while concluding that being female was associated with reduced frequency of alcohol use or alcohol-related problems, marijuana use, and sexual and other risk-taking scores [33]. She later evaluated the association between energy drink use and the strength of identification with the toxic jock syndrome, a risk-taking identity among college students. While predictive of energy drink use among male students, it was inconclusive whether the toxic jock syndrome was predictive of energy drink use among females [38]. Wimer and Levant [39] also reported on the occurrence of a risk-taking identity among college students that was associated with energy drink usage but had only a male sample. More recently, Leal and Jackson [40] investigated the associations between energy drink consumption and soft and hard drug use, and the mediating effect of age, gender and race. They found that energy drink use was significantly associated with increased soft drug use, an effect that was strongest among younger females and older males. The impact of gender on the relationship between energy drink use and risky behaviours remains an under-researched area.

In Trinidad and Tobago, among young persons the use of EDs is prevalent. In a representative sample of 1994 full- and part-time students from eight universities and colleges in Trinidad and Tobago, 86.2% of students had ever used an ED. The current prevalence of ED use, defined as use within the previous month, was 38% and use was not excessive, with most students using no more than one ED in a single sitting, five or less times per month [5]. In Trinidad and Tobago pre-mixed AmEDs are not available but “rum and red bull” refers generically to the use of alcohol mixed with energy drinks in a glass, that achieves the same effect as AmEDs. There has been no examination of the relationship between energy drink use and risky behaviours in this population.

Using the same sample, this paper will explore whether use of EDs is associated with other risk behaviours among college students in Trinidad and Tobago, and seeks to contribute to the literature by exploring the moderating effects of gender on these relationships. The study also explores whether students who combine alcohol and EDs belong to a sub-group with higher risk across a spectrum of behaviours. This would have implications for the early identification of students with high risk behaviours in need of public health prevention interventions.

## 2. Materials and Methods 

### Student Survey

The methodology has been previously published [5]. The study utilized a cross-sectional survey of students in all tertiary institutions in Trinidad and Tobago. Approval was obtained from the Ethics Committee of the Faculty of Medical Sciences, the University of the West Indies (UWI), St. Augustine campus. Eight of nine tertiary institutions in the country agreed to participate.

A de novo questionnaire collected data including student demographics, energy drink consumption patterns, alcohol consumption, and risky behaviours, including binge drinking, weekly drunkenness, marijuana use, violent/aggressive behaviours, dangerous behavior on a dare, serious physical fights, seatbelt omission, unprotected sex and number of sexual partners. The selection of questionnaire items was guided by the findings from a focus group of 15 randomly chosen students in the Faculty of Medical Sciences at the University of the West Indies (UWI). The 25-item questionnaire was piloted among 20 random students on the UWI campus, and adapted for clarity of instructions. 

A convenience sample was utilized. Quota sampling based on student enrollment ensured adequate representation of each institution. A representative number of students per faculty/department for each institution was determined using student census data in each faculty/department. Using lists of classes, timetables and class sizes, students were recruited from selected classes on different days of the week and times of day. The sample comprised 1994 full- and part-time students enrolled in all departments of the eight institutions that agreed to participate. Students were informed of the purpose of the study and anonymously completed the self-administered questionnaire. There was an 85% response rate. The only exclusion criterion was the previous completion of the questionnaire. The full questionnaire has been previously published [5].

The analyses were conducted using PASW Statistics version 19 (IBM, Armonk, NY, USA). Descriptive statistics in the form of frequencies were computed for the variables of interest. Correlation analysis examined the association between and among use of energy drinks, use of alcohol and risky behaviours. A risky behavior index was computed by adding students’ response of “yes” for all risky behaviours. This included nine (9) risky behaviors. Participants’ responses ranged from 0–2. A score of zero was given if they had never participated in the risky behavior, a score of 1 if they participated a few times (1–3 times) and score of 2 if they participated in the risky behavior 4 or more times. The lowest score a participant could get on the risky behavior index is 0 and the highest score is 18. The risky behavior index was found to be reliable (9 items; α = 0.71). Participants’ with higher values on this index indicated that they engaged in more risky behaviors. 

Risky substance use was computed by adding the scores on measures for binge drinking, weekly drunkenness, driving under the influence and marijuana use. Risky substance use was found to be reliable (4 items; α = 0.70). Non-sexual risk was computed by adding scores on measures for, violent or aggressive behavior, dangerous behavior on a dare, serious physical fights and no use of seatbelt. Cronbach’s alphas for non-sexual risk was (4 items; α = 0.57). Risky sexual behavior was computed by adding the scores on measures for unprotected sex and number of sex partners. Cronbach’s alphas for sexual risk was (2 items; α = 0.57). Though the latter alphas are below 0.70, Kline (1999) notes that when using psychological or social constructs as opposed to cognitive tests, alpha values of even below 0.7 may be accepted because these constructs are diverse [41].

A standard multiple regression was performed with risky behaviours as the criterion and predictor variables were, age, gender, use of alcohol mixed with energy drinks (in one drink), use of alcohol as well as energy drinks (separate), use of alcohol only, and use of ED drinks only. Each of the predictor variables with the exception of the demographic variables were centered and multiplied by gender to form an interaction term or variable, and included as predictors. *T*-tests were used to observe whether there were mean differences in risky behaviours based on use of the combination of energy drinks and alcohol. Between-subjects factorial analysis of variance was used to determine if gender had a moderating or interaction effect on the relation between combined ED and alcohol use and risky behaviours, as well as the relationship between intensity of ED use and risky behaviours. Data was analysed using these descriptive and inferential statistics and was guided by the specific objectives of the study.

## 3. Results

### 3.1. Demographics

Among the sample of 1994 respondents 66% were female. The mean age for the sample was 25 years, 22% were in the 19 and under age group, 58% in the 20–29 group, 12% in the 30–39 group and 8% in the 40 and older group. 

### 3.2. Descriptives and Frequencies

Among the sample of 1994 students 38% indicated that they consume energy drinks at present. Forty eight percent (48%) said they had used energy drinks before but were not currently using and 14% said they never used energy drinks before. Among male participants, 44% indicated that they were current users of energy drinks while 35% of female participants were current users. A chi-square test was performed and found that males were significantly more likely to be current users of EDs, *χ*^2^(1, *N* = 1989) = 15.02, *p* < 0.001. When asked the reason for consuming energy drinks, multiple response frequency analysis showed that the most popular response was use for energy boost (26%), followed by for staying awake (24%), studying or major project (21%), then for sports (12%), mixing with alcohol (12%), mental enhancer (4%) then for treating hangovers (1%).

**Figure 1:**
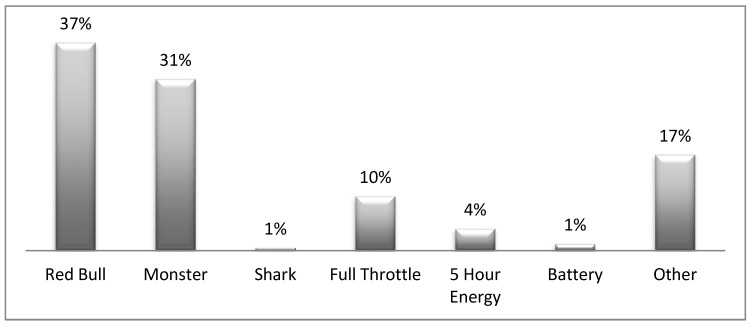
Energy drinks mainly consumed.

The type of energy drinks most frequently consumed by the sample is Red Bull as 37% indicated the same. See Figure 1 for frequency of use for other energy drinks.

Of those who responded (*N* = 1963) to their use of alcohol and energy drinks, 27% indicated that they consumed both energy drinks and alcohol, 33% indicated that they consumed only alcohol, 11% said they consumed only energy drinks and 29% indicated that they consumed neither energy drinks nor alcohol. 

**Figure 2:**
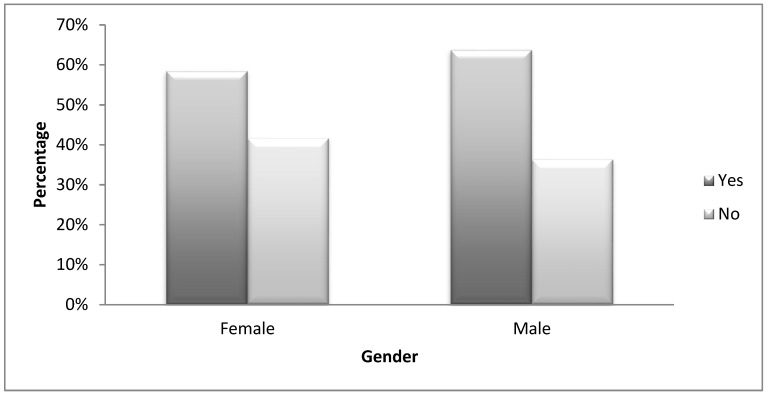
Use of alcohol within gender.

Three hundred and eighty-three (383) or 19% of students indicated that they use energy drinks to mix with alcohol while partying; 60% of students said they used alcohol. The within gender percentages show that proportionately, more males indicated that they use alcohol. This was not statistically significant *χ*^2^(1, *N* = 378) = 0.40, *p* = 0.53. See Figure 2. 

### 3.3. Use of Energy Drinks and Risky Behaviours

#### 3.3.1. Correlations

Correlation analysis indicate that males were more likely than females to use alcohol and energy drinks together, *r_pb_* (1989) = 0.07, *p* < 0.05.

There was a significant correlation between use of combined energy drinks and alcohol and risky behaviors index *r* (1876) = 0.26, *p* < 0.001. Students who combined energy drinks with alcohol had a higher index of risky behaviors. Independent samples *t*-test also showed a significant difference in the scores on risky behaviours for students who use EDs (*M* = 3.92, *SD* = 3.55) and those who do not (*M* = 2.08, *SD* = 2.57); *t* (484.10) = −9.85, *p* = 0.000.

Spearman’s correlation analysis showed that students who reported current use of energy drinks were significantly more likely to report other risky behaviours. There was however no significant association between current energy drink use and not using a seatbelt, as well as for serious physical fights (see Table 1).

Both males and females who were current users of energy drinks were more likely to engage in risky behaviours. The significant correlations for males and females are shown in Table 1.

Of current energy drink non-users, 5.8% had more than one sexual partner in the past month compared to 12.5% of current users. Correlation analysis indicate that users of EDs had more sexual partners per month than non-users, *r* (1911) = 0.173, *p* = 0.000.

#### 3.3.2. Multiple Regression

A standard multiple regression was performed with risky behaviours as the criterion and predictor variables were age, gender, use of alcohol mixed with energy drinks (in one drink), use of alcohol as well as energy drinks (separate), use of alcohol only, and use of EDs only. Each of the predictor variables with the exception of the demographic variables were centered and multiplied by gender to form an interaction term or variable, and included as predictors. *Multiple R* for regression was significantly different from zero, F(10, 1829) = 40.68, *p* <.001, with *R*^2^ at 0.18 and 95% confidence limits from 0.15 to 0.21. The ten predictors in combination contributed another 0.06 in shared variability. Altogether, 18% of the variability in risky behaviours was predicted by knowing scores of these ten predictors. The size and direction of the relationships suggests that students who are males, those who drank alcohol only, who use energy drinks mixed with alcohol, as well as those who consume both alcohol and ED (though not mixed), are more likely to engage in risky behaviours. Gender seems to be the strongest predictor of risky behaviors with males having more risk behaviors. There is a significant interaction of alcohol and ED use with gender, indicating that males who consume both drinks were the ones more likely to engage in risky behavior. Table 2 displays the unstandardized regression coefficients *(b*), the standardized coefficient (*β*), the semi-partial correlation (*sr*^2^), and *R*^2^. The other interaction terms were not significant. When the university campus (faith-based or not) was included as a predictor, there was no difference to the outcome of the analysis. 

#### 3.3.3. Factorial ANOVA

##### One-way ANOVA

A one-way between-participants analysis of variance (ANOVA) was conducted to investigate whether there were differences in engaging in risky behaviors based on the use of alcohol and energy drinks. The sample was divided based on whether students drank: (1) a combination of alcohol and energy drinks (2) energy drinks only (3) alcohol only or (4) neither alcohol nor energy drinks. 

ANOVA indicated that there was a significant effect of use of drinks on risky behaviour F(3, 1867) = 51.40, *p* < 0.0001, *η_p_*^2^ = 0.08, *MSE* = 7.47 All possible post hoc pairwise comparisons were conducting using Bonferroni correction. There was a difference in scores for risky behaviour between students who used both energy drinks and alcohol and those who only drank alcohol, *t* (1147) = 4.53, *p* < 0.001. There was also a significant difference between students who used both drinks and those who use only energy drinks, *t* (709) = 7.42, *p* < 0.001, and those who drank neither energy drinks nor alcohol *t* (1045) = 11.64, *p* < 0.001. Further, risky behavior scores for students who drank only alcohol were different from that of students who drank only energy drinks, *t* (822) = 4.34, *p* < 0.001. 

**Figure 3:**
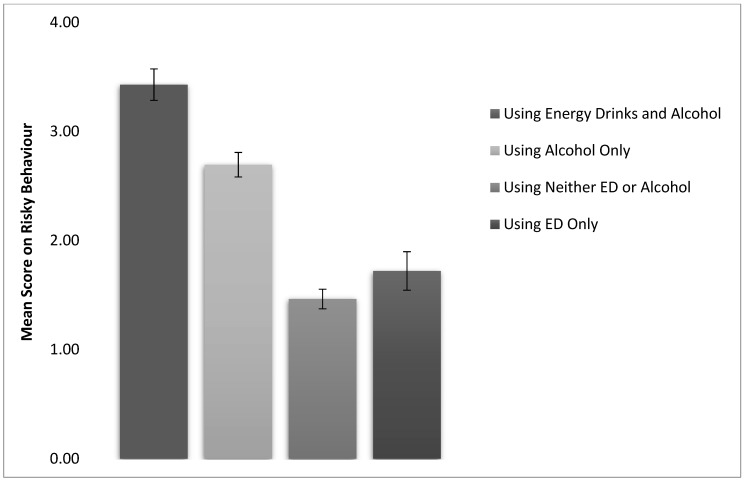
Mean scores for risky behavior based on use of drinks.

Overall mean scores for risky behaviours were significantly higher for students who used both energy drinks and alcohol (*M* = 3.43, *SD* = 3.27) than for those who drank only alcohol (*M* = 2.70, *SD* = 2.82), those who drank only energy drinks (*M* = 1.72, *SD* = 2.46) and those who drank neither drinks (*M* = 1.46, *SD* = 2.06). Moreover, those who drank only alcohol engaged in more risky behaviors than those who drank only energy drinks. Furthermore, risky behaviour scores were significantly lower for students who drank neither alcohol nor energy drinks than for all the other groups (see Figure 3), with the exception of students who only drank energy drinks, there no significant difference between the two groups, *t* (1158) = 1.12, *p* = 1.00.

To explore the main and interaction effects of gender, use of alcohol and intensity of energy drinks use on risky behaviours, a 3-way between-subjects Analysis of variance was conducted. A 2 (Intensity ED use: 1–5 ED per month, 6 or more ED per month) × 2 (use of alcohol: Yes, No) × 2 (gender: male, female) independent ANOVA was conducted. There was a significant intensity of ED use main effect, F(1, 1519) = 10.26, *p* = 0.001. Students who drank an average of 6 or more EDs per month had higher scores on the risky behaviour index (*M* = 3.80, *SD* = 3.79) than those who drank less (*M* = 2.44, *SD* = 2.78).

There was also a significant alcohol use main effect, F(1, 1519) = 58.61, *p* = 0.000. Students who drank alcohol had a significantly higher score on the risky behavior index (*M* = 3.11, *SD* = 3.13) than those who did not drink (*M* = 1.63, *SD* = 2.30). 

There was a significant gender main effect, F(1, 1519) = 74.36, *p* = 0.000. Males had a significantly higher score on the risky behavior index than females. Males therefore engaged in more risky behaviors (*M* = 3.87, *SD* = 3.71) than females (*M* = 1.94, *SD* = 2.19).

**Figure 4:**
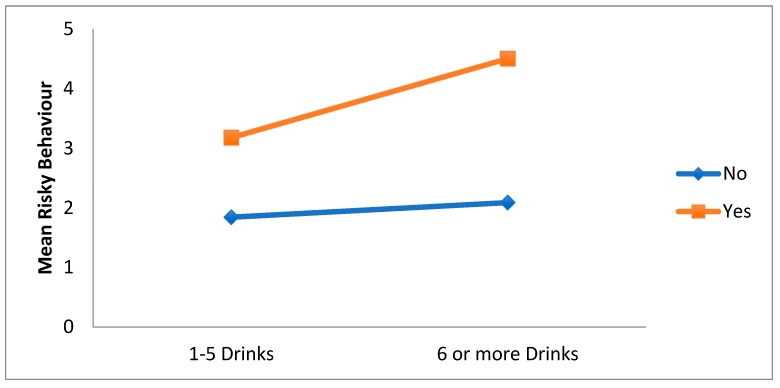
Interaction effect of alcohol use (yes/no) on the relationship between intensity of energy drinks use (1–5/6 or more drinks) and overall risky behavior.

There was a significant alcohol × intensity ED use interaction effect. The main effect for intensity of energy drinks was qualified by a significant alcohol interaction, F(1, 1519) = 4.87, *p* = 0.028. As can be seen in Figure 4, for both categories of intensity of ED use, risky behaviours are higher for those who use alcohol than for those who do not, with those who consume alcohol recording the highest risky behaviors score. Thus, increased risky behaviours seem to occur more for those who use on average 6 or more energy drinks a month and who also drink alcohol. 

**Figure 5:**
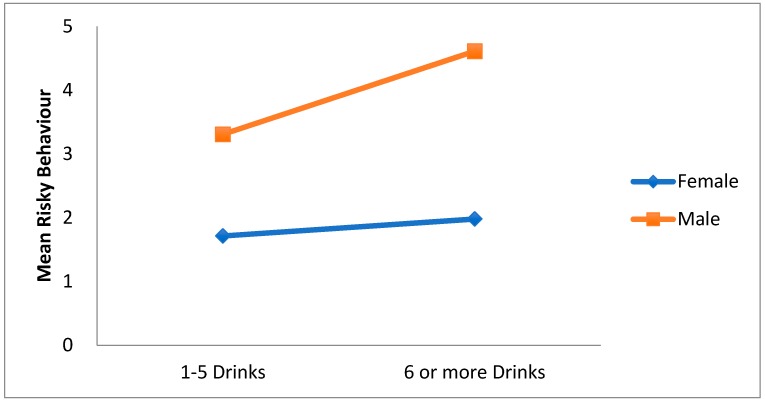
Interaction effect of gender on the relationship between intensity of energy drinks (1–5/6 or more drinks) and overall risky behavior.

There was also a significant intensity of ED use x gender interaction effect. F(1, 1519) = 4.48, *p* = 0.034. As can be seen in Figure 5, for both categories of intensity of ED use, risky behaviour is higher for males than for females. Thus, increased risky behaviour seem to occur more for men who consume an average of 6 or more energy drinks a month.

There was no significant alcohol use x gender interaction F(1, 1519) = 0.77, *p* = 0.389. There was also no significant 3-way interaction of Intensity of ED use x alcohol use x gender F(1, 1519) = 0.04, *p* = 0.850.

To investigate the influence of gender, the intensity of ED use (average EDs consumed per month) and use of alcohol on three categories of risky behaviors (risky substance use, risky sex, and non-sexual risk) a series of 3-way between-subjects factorial ANOVAs were conducted.

For risky substance use, a 2 (Intensity ED use: 1–5 ED per month, 6 or more ED per month) × 2 (use of alcohol: Yes, No) × 2 (gender: male, female) independent ANOVA was conducted. There was a significant gender main effect, F(1, 1516) = 57.57, *p* = 0.000. Males had a significantly higher score on the risky substance use index than females. Males therefore engaged in more risky substance use (*M* = 1.74, *SD* = 2.07) than females (*M* = 0.66, *SD* = 1.19). 

There was, also, a significant intensity of ED use main effect, F(1, 1516) = 11.23, *p* = 0.001. Students who drank an average of 6 or more EDs per month had higher scores on the risky substance use index (*M* = 1.68, *SD* = 2.04) than those who drank less (*M* = 0.94, *SD* = 1.55). 

There was, also, a significant alcohol use main effect, F(1, 1516) = 106.49, *p* = 0.001. Students who drank alcohol had higher scores on the risky substance use index (*M* = 1.42, *SD* = 1.78) than those who did not (*M* = 0.27, *SD* = 0.92). 

There was no significant interaction effect of gender on the relationship between intensity of ED use and risky substance use F(1, 1516) = 1.23, *p* = 0.267. There was no significant interaction effect of alcohol on the relationship between intensity of ED use and risky substance use F(1, 1516) = 1.16, *p* = 0.282. There was also no significant interaction effect of gender on the relationship between alcohol use and risky substance use F(1, 1516) = 3.29, *p* = 0.070. There was no significant 3-way interaction F(1, 1516) = 2.72, *p* = 0.01.

For risky sex, a 2 (Intensity ED use: 1–5 ED per month, 6 or more ED per month) × 2 (use of alcohol: Yes, No) × 2 (gender: male, female) independent ANOVA was conducted. There was a significant gender main effect, F(1, 1565) = 19.03, *p* = 0.000. Males had a significantly higher score on the risky sex index than females. Males therefore engaged in more risky sexual behaviour (*M* = 1.37, *SD* = 1.35) than females (*M* = 0.95, *SD* = 1.15). 

There was also a significant intensity of ED use main effect, F(1, 1565) = 12.72, *p* = 0.000. Students who drank an average of 6 or more EDs per month scored higher on the risky sexual behavior index (*M* = 1.51, *SD* = 1.35) than those who drank less (*M* = 1.04, *SD* = 1.21). 

There was, also, a significant alcohol use main effect, F(1, 1565) = 23.82, *p* = 0.000. Students who drank alcohol had higher scores on the risky substance use index (*M* = 1.27, *SD* = 1.26) than those who did not (*M* = 0.77, *SD* = 1.12). 

There was no significant interaction effect of gender on the relationship between intensity of ED use and risky sex F(1, 1565) = 1.56, *p* = 0.212. There was no significant interaction effect of alcohol on the relationship between intensity of ED use and risky sex F(1, 1565) = 0.15, *p* = 0.70. There was also no significant interaction effect of gender on the relationship between alcohol use and risky sex F(1, 1565) = 0.33, *p* = 0.56. There was no significant 3-way interaction F(1, 1565) = 0.01, *p* = 0.91.

For non-sexual risk, a 2 (Intensity ED use: 1–5 ED per month, 6 or more ED per month) × 2 (use of alcohol: Yes, No) × 2 (gender: male, female) independent ANOVA was conducted. There was a significant gender main effect, F(1, 1515) = 60.50, *p* = 0.000. Males had a significantly higher score on the non-sexual risk index than females. Males therefore engaged in more non-sexual risk (*M* = 1.47, *SD* = 1.82) than females (*M* = 0.75, *SD* = 1.10). 

There was, also, a significant intensity of ED main effect, F(1, 1515) = 5.72, *p* = 0.017. Students who drank an average of 6 or more EDs per month had higher scores on the non-sexual risk index (*M* = 1.44, *SD* = 1.86) than those who drank less (*M* = 0.94, *SD* = 1.35). 

**Figure 6:**
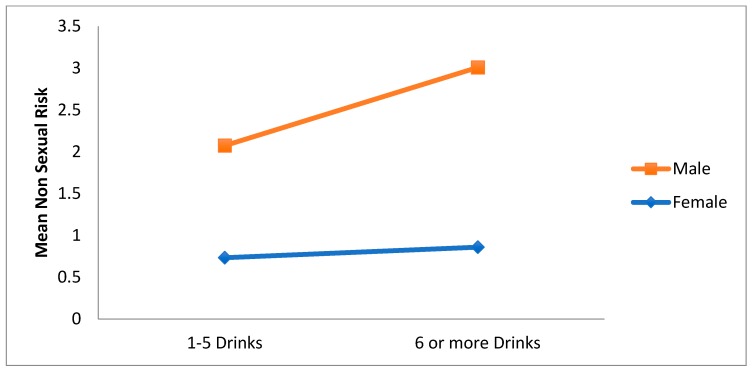
Interaction effect of gender on the relationship between intensity of ED use and non-sexual risk.

The main effect for intensity of ED use was qualified by a significant gender x intensity interaction, F(1, 1515) = 5.80, *p* = 0.016. As can be seen in Figure 6, for both categories of intensity of ED use, non-sexual risk is higher for males than for females. Thus, increased non-sexual risk seem to occur more for men who consume an average of 6 or more energy drinks a month.

**Figure 7:**
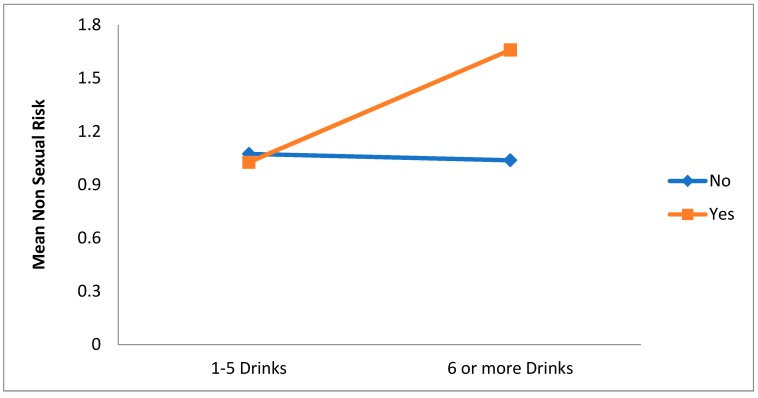
Interaction effect of alcohol use on the relationship between intensity of ED use and non-sexual risk.

The main effect for intensity ED use was also qualified by a significant alcohol × intensity of ED use interaction, F(1, 1515) = 7.21, *p* = 0.007. As can be seen in Figure 7, for persons who drink 1–5 energy drinks and who do not drink alcohol, non-sexual risk is slightly higher than for those in the same category who use alcohol. However for those who drink 6 or more energy drinks, non-sexual risk is significantly higher than for those who drink alcohol than for those who do not. Thus, increased non-sexual risk seem to occur more for those who use alcohol and have an average of 6 or more energy drinks a month.

There was also no significant interaction effect of gender on the relationship between alcohol use and non-sexual risk F(1, 1515) = 0.02, *p* = 0.90. There was no significant 3-way interaction F(1, 1515) = 1.65, *p* = 0.20.

## 4. Discussion

This study explored, for the first time, the relationship between energy drink use and indices of risky behaviours in the English-speaking Caribbean. The findings showed that among university students in Trinidad and Tobago there was a consistent association between energy drink consumption and all indices of risky behaviours that were investigated, including harmful substance use, risky sexual behaviours and risky non-sexual behaviours such as violence, acting on a dare, fights and seat belt omission. Greater use of energy drinks was significantly predictive of riskier behaviours. 

This is consistent with previous reports among adolescents and young persons. Energy drink users in Australia and Canada were more likely to report heavier alcohol consumption, and use of cigarettes, illicit drugs and non-medicinal prescription drugs, compared to non-users [3,20]. Similarly, college students and young adults in the USA were likely to participate in risky and delinquent behaviours more often than non-ED users [6,32,34]. In Denmark in 2014, where the prevalence of ED use was noted as lower than other European countries, young persons who used alcohol and cigarettes were more inclined to consume energy drinks [4]. 

There was a clear relationship between the intensity of energy drink use and engagement in risky behaviours. This was also reported by Peacock et al. [31], Miller [33] and Kaminer [35] all of whom found that risk taking behaviours were positively associated with frequency of energy drink consumption. Woolsey et al. [37] found that the frequency of energy drink use was a significant predictor of the illicit use of prescription stimulants. In this study intensity of ED use was measured by the average number of drinks consumed per month but the direct correlation with risk taking behaviours was the same.

It has been hypothesized but not conclusively demonstrated in other studies [38] that the relationship between energy drink consumption and risk taking would be stronger for males than females. This was found to be the case in this study. While males were significantly more likely to consume energy drinks, male and female university students in Trinidad and Tobago were both at significantly increased risk of harmful behaviours with increasing use of energy drinks. The correlation was however stronger among males. 

Unlike many others, this study collected data that afforded the comparison of differences in risky behaviour based on whether students used alcohol in combination with energy drinks. The use of alcohol, with or without energy drinks was a significant predictor of risky behaviours. A minority of students (22%) drank alcohol with energy drinks as mixer. This is consistent with the 19–27% prevalence of AmED use reported among college students by others [6,20,21,28]. While energy drink users represented a group of students in Trinidad and Tobago who were more likely to engage in risky behaviours, this sub-group of students who mixed energy drinks and alcohol in a setting where AmEDs are not available pre-packaged, were at even greater risk of health compromising behaviours, an observation that held for both male and female students, but was stronger for males. Interestingly however, the findings suggested that there was an increased risk of health compromising behaviours regardless of whether the student used both types of drink independently, or in a mixed cocktail. Caution is taken in this interpretation since data was not collected on the quantity of alcohol consumed, nor was it determined how many students using both alcohol and EDs did so in the same sitting, a scenario that would be akin to the use of AmEDs. There is therefore no measure of whether the occurrence of risky behaviours was related to the intensity of alcohol use, or whether there was mixing of the two types of drinks. Nonetheless, the highest risky behaviour scores in all of the calculated measures were found among students who drank alcohol and reported a high intensity of energy drink usage. This was greatest for male students. Students who reported drinking only energy drinks had significantly lower risky behaviour scores than those who drank alcohol only, or both types of drinks. 

Even though there was no significant interaction effect of gender on the relationship between ED use or use of EDs in combination with alcohol and overall risky behaviour, the present study found no gender distinction as it related to the association between intensity of energy drink use and increased alcohol-related harm (measured by binge drinking, frequent intoxication and drink-driving), and risky sexual behaviours (measured by multiple partnering and unprotected sex). Miller suggested in her 2008 report that energy drink use may be a potential predictor of a toxic jock identity comprising a sport-related identity, endorsement of masculinity norms and risk taking that was observed among both male and female college students in the USA [38]. She found a positive association between frequency of energy drink use and strength of identification as a jock, a relationship that was mediated by both masculine norms and risk-taking behavior. Wimer and Levant [39] also identified endorsement of traditional masculinity ideology as a significant predictor of energy drink use, a relationship that was moderated by self-identification as a jock. It is noteworthy therefore that in the present study both males and females who used energy drinks were equally likely to engage specifically in high risk behaviours of alcohol excess and sexual risk, core component elements of the stereotype of masculinity portrayed in the local media and described by Lewis in his treatise on gender and performativity in the Caribbean [42]. This is an area that warrants further exploration as it suggests that in the Caribbean risky behaviours associated with energy drinks may be related more to the individual and the adoption of the dominant masculine norms than to the energy drink per se. Rather use of energy drinks may be associated with a high-risk identity, as described by Miller [38] and Wimer and Levant [41], that centres around hypermasculinity and risk taking. 

It has been found globally that young people who engage in one risky behaviour often engage in several other kinds of risky behaviour as well. The findings of this Caribbean study, like previous works done in other world regions, suggest that a high intensity of energy drink use, and in particular when combined with alcohol is one such expression of risky behaviour. Findings support the suggestion of Kaminer [35] that frequent/intense energy drink use may be a marker for high risk behaviours and may therefore be a useful screening indicator to identify students at risk for substance use, sexual risk taking and other problem behaviors. Others have even suggested that energy drink use may be an indicator of possible escalation in legal and illicit drug use [37,39], and may represent the beginning of drug use progression, playing a significant role in the escalation of soft drug use and subsequent progression to hard drug use by way of soft drug use [40,43]. This would be of particular usefulness as a fairly stable indicator of health risk among young persons in Trinidad and Tobago where the effect of risky behaviours on the health of youth is a source of concern. A 2010 World Bank report estimated that 45–80% of the youth population in Latin America and the Caribbean are at risk of engaging in negative behaviours with or without serious consequences [44]. Caribbean youth begin alcohol use earlier than in other world regions [45], and it has been reported that 31% of university students in Trinidad and Tobago binge drink [46]. Reviews have generally noted the high lifetime prevalence of substance use and risky sexual behaviours among youth, and the associated significant health effects manifested as mental illness (which would include substance use disorders), violence and juvenile delinquency, teenaged pregnancy and sexually transmitted infections, including HIV [44,47]. HIV seroprevalence in Trinidad and Tobago was reported at 1.6% in 2014 with young adults accounting for 12% of new cases; 64% of these were among 15–49 year olds [48]. During 2003 to 2004 the highest incidence of HIV was in the 15–24 year and 25 to 34 year age groups among females [49].

Energy drink consumption can result in adverse health effects in adolescents and young adults through the direct effects of caffeine and other ingredients [5,8,10]. Energy drink use also appears to pose a significant threat to youth health through increased association with alcohol and other drug use, and other risky behaviours. Early identification of high risk youth and young adults has to be a priority for public health professionals in Trinidad and Tobago. Based on the findings of this study, the intensity of use of EDs and whether they are combined with alcohol may serve as a marker for the early identification of risky health behaviours. In assessing and researching health behaviours among adolescents and young adults, enquiry about the amount of energy drinks used and concomitant alcohol use may afford a discriminating and easily elucidated index of high risk behaviours that allows for easier and earlier implementation of primary and secondary prevention programmes. Further studies will be needed to clarify whether there is an alcohol dose-dependent and/or interactive effect.

This study assumes a general representation of university students in Trinidad and Tobago but findings cannot be generalized to young persons who are not in tertiary education. One strength of the study is the wide range of data that was collected and allowed for exploration by gender, and comparative analysis of students who used one, both or neither of energy drinks and alcohol, but a major limitation was the absence of data on the quantity of alcohol used by students. Another major limitation of the study was in the reliability of the measures of risky behaviour that were developed. Even though some leeway is given for measures with social constructs, two of the risk measures had less than acceptable alpha reliability coefficients and for the other two the reliability coefficients were just acceptable. The study might have benefitted from a more thorough interrogation of the extent and context of use of energy drinks, alcohol, and combined EDs and alcohol. Further studies should seek a clearer understanding of the demographic, contextual and social factors influencing the relationship between use of energy drinks and other health-risk behaviours, and the underlying personality and motivational risk factors. Particularly, further studies should explore for the possibility of a high-risk identity, predicated on masculinity ideology that may underlie energy drink use and negative health-related behaviours in Trinidad and Tobago. An understanding of the information elucidated from further investigation will be of great importance when developing primary and secondary prevention interventions for young persons, and will have significant implications for public health and public policy surrounding energy drinks in Trinidad and Tobago. 

## 5. Conclusions

This study explored the relationship between energy drink use and risky behaviours for the first time in a Caribbean population. Findings showed that among undergraduate students in Trinidad and Tobago, intensity of energy drink usage was significantly correlated with risky behaviours, regardless of gender. Consistent with reports from other world regions, male gender, high intensity of energy drink use, and combined use of alcohol and energy drinks were the strongest predictors of risky behaviours. In Trinidad and Tobago there is concern over the high prevalence of public health issues such as alcohol use disorders, teenaged pregnancy and sexually transmitted infections, that are related to alcohol use and sexual risk taking among young adults. Intensity of energy drink use, especially in combination with alcohol, may be a useful screening indicator of health risk, that will allow for the early identification of, and intervention with young adults engaged in risky health behaviours.

## Figures and Tables

**Table 1:** Spearman’s Rho correlation coefficients for risky behaviors and ED consumption.

Risky Behaviors	^1^ Do you Consume Energy Drinks (Yes/No)	
	Males	Females
Binge drinking (>5 drinks in one sitting)	0.192 **	0.135 **
Weekly drunkenness	0.138 **	0.109 **
Driving under the influence of alcohol	0.139 **	0.084 **
Marijuana use	0.092 *	0.027
Violent/aggressive behavioiur	0.088 *	0.057 *
Dangerous behaviour on a dare	0.101 *	0.068 *
Serious physical fight	0.068	0.036
No use of a seatbelt	0.045	−0.028
Unprotected sex	0.103 **	0.035

** Correlation is significant at the 0.01 level (2-tailed); * Correlation is significant at the 0.05 level (2-tailed); ^1^ Yes = 1, No = 0.

**Table 2:** Standard multiple regression of predictors on risky behaviour.

Predictor Variables	Outcome Variable			
	Risky Behaviours *N* = 1840			
	*b*	*SE b*	*β*	*sr* ^2^
Gender	1.53	0.13	0.26 ***	0.06
Age	−0.01	0.01	−0.02	0.00
Use of alcohol combined with energy drinks (in one drink)	1.62	0.16	0.16 ***	0.02
Use of alcohol as well as energy drinks (separate)	1.38	0.17	0.22 ***	0.03
Use of Alcohol only	0.93	0.16	0.16 ***	0.02
Use of Energy Drinks only	−0.07	0.27	−0.01	0.00
Use of alcohol combined with energy drinks (in one drink) * Gender	0.58	0.33	0.04	0.00
Use of alcohol and energy drinks separate * Gender	1.02	0.36	0.08 **	0.00
Use of Alcohol only * Gender	0.43	0.34	0.03	0.00
Use of Energy Drinks only * Gender	0.60	0.47	0.04	0.00

Note: *R*^2^ = 0.18; Δ*R*^2^ = 0.18; * *p* < 0.05; ** *p* < 0.01; *** *p* < 0.001; unique variability = 0.12. Gender is dummy coded Male = 1, Female = 0.
